# Supplementary material for: Disruption of a six-nucleotide miRNA motif improves PKD1 dosage and ameliorates polycystic kidney disease
Source: Nucleic Acids Res. 2026 Jan 21;54(2):gkaf1538. doi: 10.1093/nar/gkaf1538 (PMC12818905; doi:10.1093/nar/gkaf1538)
Supplement: gkaf1538_Supplemental_Files [file gkaf1538_supplemental_files.zip › Supplemental Figures with legends.pdf]

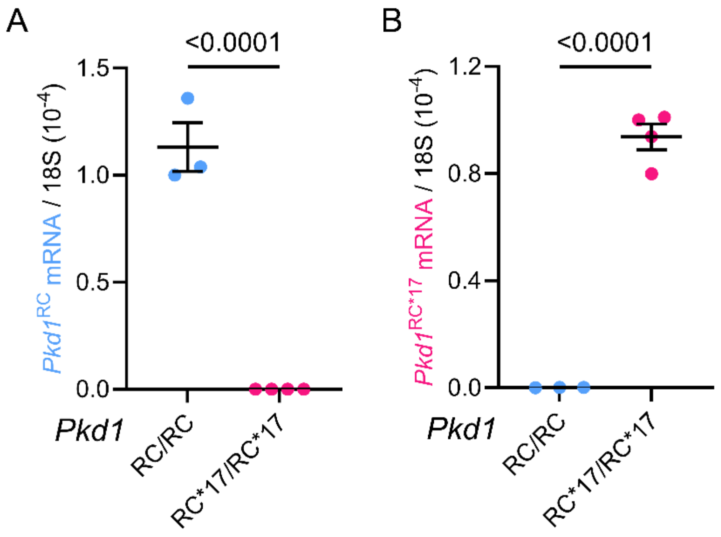

Supplementary Figure 1. Validation of allele-specific qRT-PCR primers to detect *Pkd1*$^{\mathbf{RC}}$ and *Pkd1*$^{\mathbf{RC*17}}$ alleles. A&B. qRT-PCR using RNA from primary kidney epithelial cells derived from *Pkd1*$^{RC/RC}$ and *Pkd1*$^{RC*17/RC*17}$ mice demonstrates allele-specific amplification of *Pkd1*^{RC} and *Pkd1*^{RC*17} transcripts respectively. Error bars indicate SEM. N=3, biological replicates. Statistics: Unpaired, two-tailed, t-test.

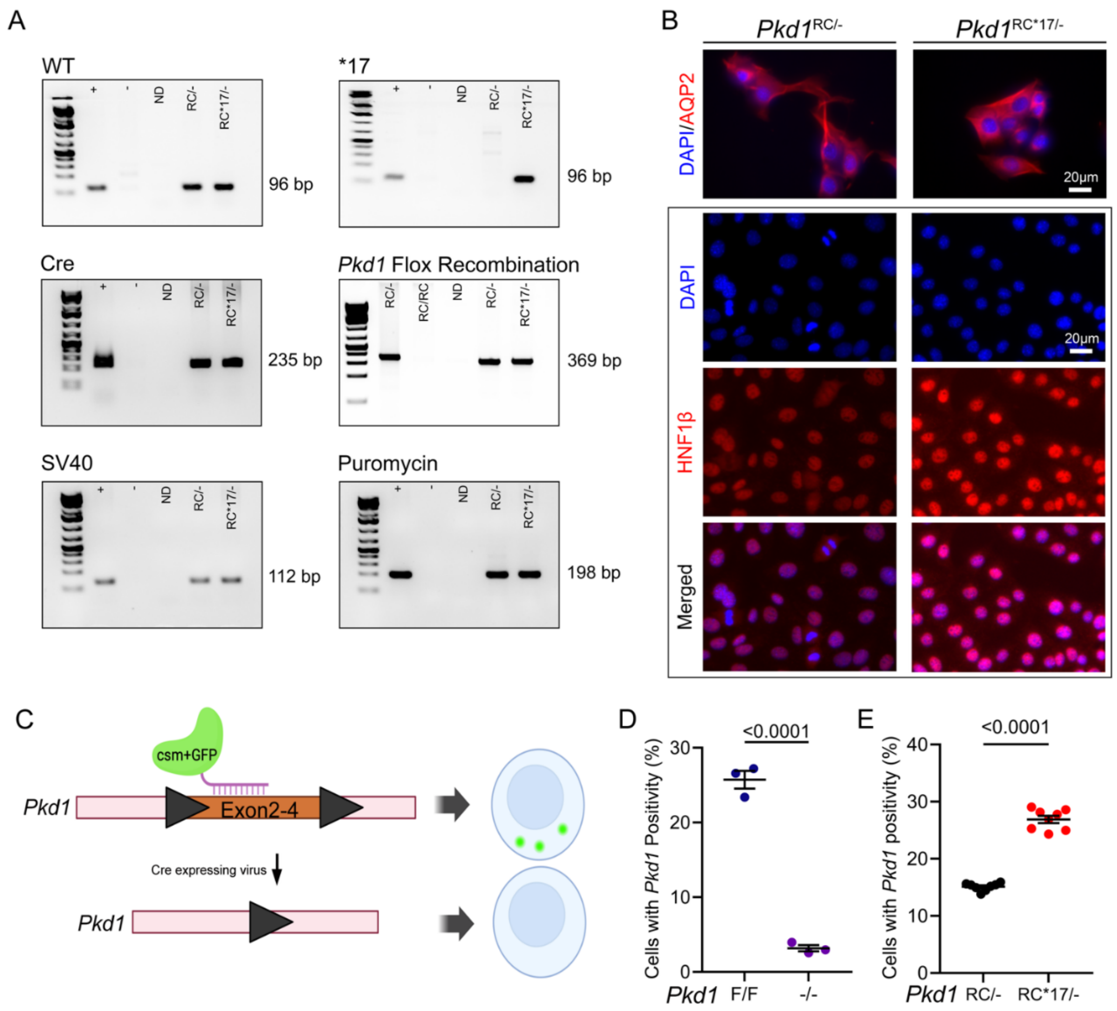

Supplementary Figure 2. Characterization of *Pkd1*$^{RC/-}$ and *Pkd1*$^{RC*17/-}$ cell lines. Kidney epithelial cells enriched for DBA were isolated from 10-day-old *Ksp*$^{Cre+}$;*Pkd1*$^{RC/F}$ and *Ksp*$^{Cre+}$;*Pkd1*$^{RC*17/F}$ mice kidneys. Single clones were isolated for subsequent analysis. **A.** Gel electrophoresis confirmed genotypes of *Pkd1*^{RC} (WT 3'UTR) and *Pkd1*^{RC*17} (*17 3'UTR) alleles in *Pkd1*$^{RC/-}$ and *Pkd1*$^{RC*17/-}$ cell lines. Presence of *Ksp*Cre transgene and successful recombination of *Pkd1* is shown. Immortalization with SV40 is confirmed by the presence of SV40 and puromycin cassette. **B.** Immunofluorescence demonstrated presence of collecting duct marker aquaporin 2 (AQP2) and epithelial marker HNF1B. **C-D.** Schematic illustration of the CRISPR-based live-cell RNA sensing system to detect *Pkd1* mRNA. A sgRNA was designed specifically to target *Pkd1* exons 2-4. In *Pkd1*$^{F/F}$ cells, the *Pkd1*-SgRNA directs the catalytically inactive CRISPR-Cas complex (csm-GFP) to the mRNA, resulting in fluorescent puncta. However, *Pkd1*$^{-/-}$ cells (derived from *Pkd1*$^{F/F}$ cells by infecting them with a virus expressing Cre recombinase) should demonstrate no fluorescent puncta signal with the csm-GFP-*Pkd1*-SgRNA system. Accordingly, live cell quantification demonstrates GFP puncta in *Pkd1*$^{F/F}$ cells but not *Pkd1*$^{-/-}$ cells, implying specificity of *Pkd1*-SgRNA in detecting *Pkd1* mRNA. **E.** This *Pkd1* mRNA detection system was used in *Pkd1*$^{RC*17/-}$ and *Pkd1*$^{RC/-}$ cells (shown in A-B). *Pkd1* mRNA detection was 50% higher in in *Pkd1*$^{RC*17/-}$ compared to *Pkd1*$^{RC/-}$ cells. Error bars indicate SEM. N=3, biological replicates. Statistics: Unpaired, two-tailed, t-test.

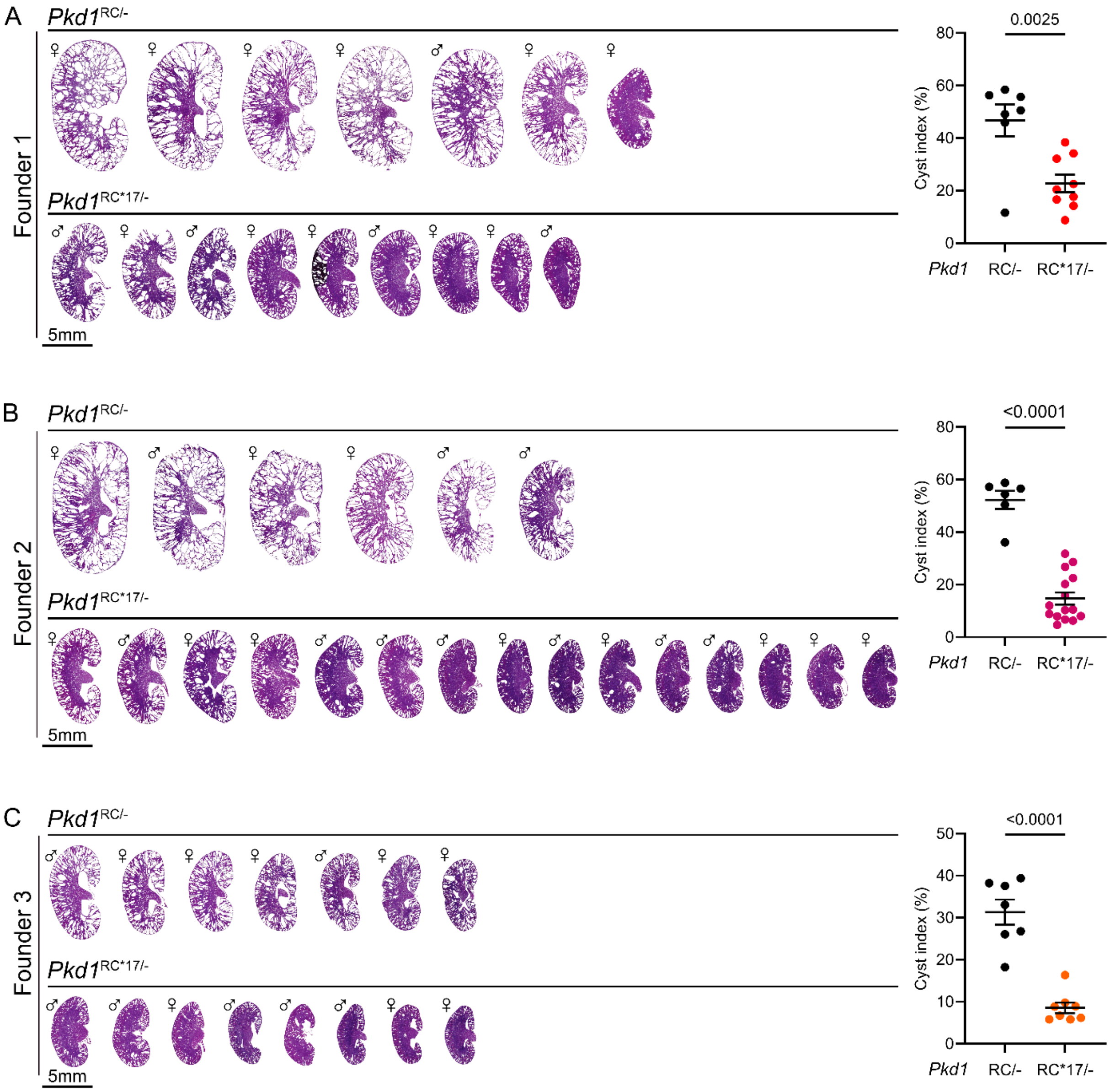

Supplementary Figure 3. Kidney section H&E and cyst indices of *Pkd1*$^{\text{RC/-}}$ and *Pkd1*$^{\text{RC*17/-}}$ mice. Gross H&E of kidney sections and the associated kidney cyst index of every 18-day-old *Pkd1*$^{\text{RC/-}}$ and *Pkd1*$^{\text{RC*17/-}}$ mouse analyzed in this study is shown. The data from Founder 1, 2, and 3 are shown in A, B, and C, respectively. Gender is indicated by symbol to the left of each kidney image. Error bars indicate SEM. Statistics: Unpaired, two-tailed, t-test.

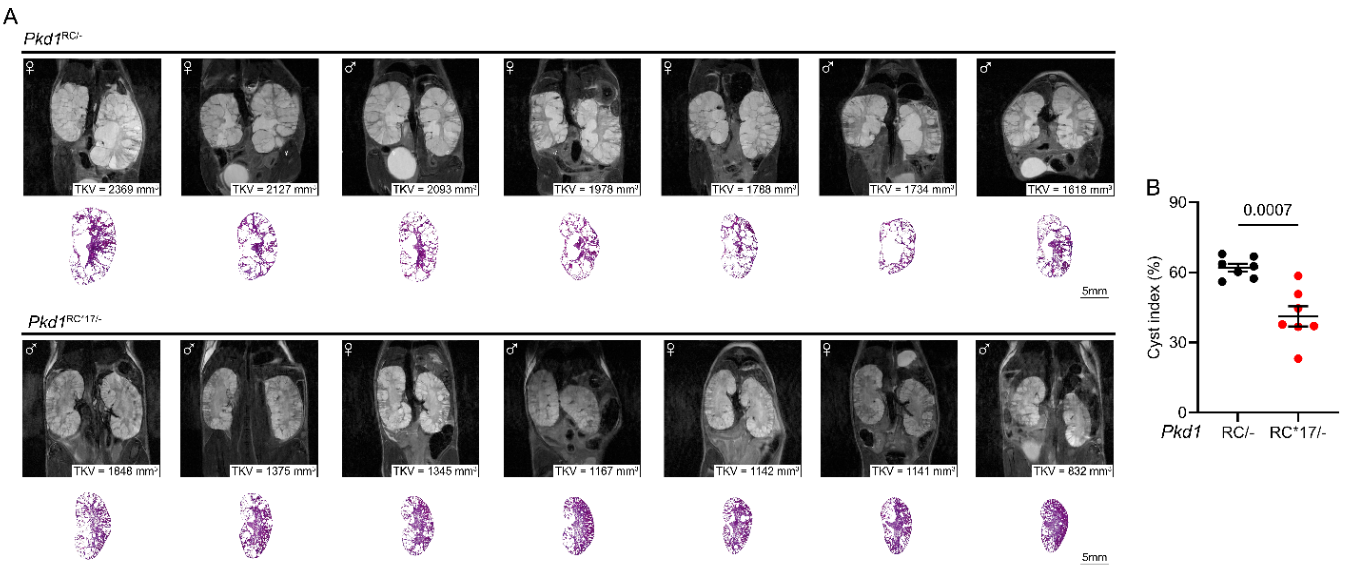

Supplementary Figure 4. MRI-based anatomical and kidney histological analysis of aged *Pkd1*$^{RC/-}$ and *Pkd1*$^{RC*17/-}$ mice. A. MRI of midline coronal section of kidneys with corresponding left kidney section H&E of every 84-day-old littermate *Pkd1*$^{RC/-}$ and *Pkd1*$^{RC*17/-}$ mice analyzed in this study is shown. Gender of each mouse is indicated in top left of each MRI image. Corresponding total kidney volume (TKV) is denoted in mm^3 in each MRI. **B.** Cyst index based on kidney section H&E in *Pkd1*$^{RC*17/-}$ kidneys compared to *Pkd1*$^{RC/-}$ kidneys is shown. Error bars indicate SEM. Statistics: Unpaired, two-tailed, t-test.

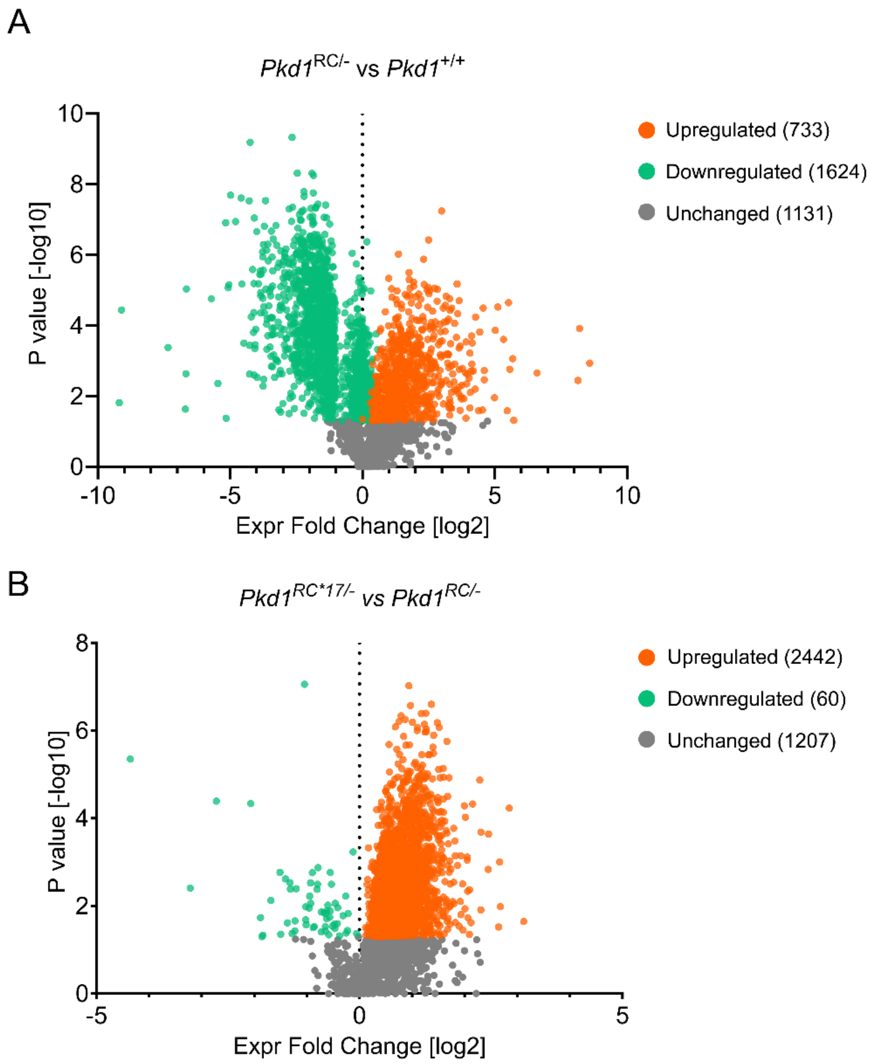

Supplementary Figure 5. Proteomic analysis of *Pkd1*$^{+/+}$, *Pkd1*$^{RC/-}$, and *Pkd1*$^{RC*17/-}$ kidneys. A&B. Volcano plot depicts change in protein expression patterns (upregulated = orange; downregulated = green; unchanged = gray) between *Pkd1*$^{+/+}$ and *Pkd1*$^{RC/-}$, and *Pkd1*^{RC} and *Pkd1*$^{RC*17/-}$ mice kidneys. $P < 0.05$ was considered significantly changed. N=3 biological replicate for each group. Statistics: Unpaired, two-tailed, t-test.

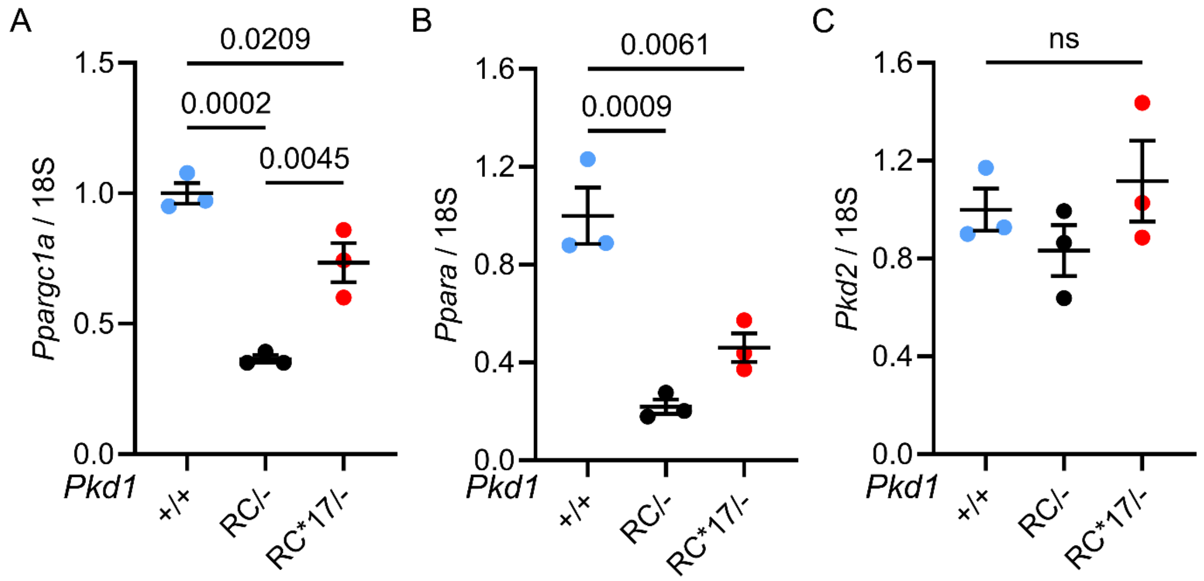

Supplementary Figure 6. qRT-PCR of additional genes involved in the c-Myc-miR-17 axis. A. Expression of *Ppargc1a*, a key regulator of mitochondrial biogenesis is reduced in *Pkd1*$^{RC/-}$ kidneys compared to *Pkd1*$^{+/+}$ kidney, but is restored in *Pkd1*$^{RC*17/-}$ kidneys. **B.** In contrast, the expression of *Ppara*, a direct target of miR-17 in the context of PKD, is not improved in *Pkd1*$^{RC*17/-}$ kidneys. **C.** The expression of *Pkd2*, another miR-17 target, was also not different between *Pkd1*$^{+/+}$, *Pkd1*$^{RC/-}$ and *Pkd1*$^{RC*17/-}$ kidneys. Error bars indicate SEM. N=3 biological replicates in all groups. Statistics: ANOVA with Tukey's.

A

Pkd1 oligo (P1)

Pkd1 3'UTR 5' CACATATGGGGCTTCT**GCACTTTA**AAAAGGCTGTGT 3'

B

PKD1 oligo (P2)

PKD1 3'UTR 5' TGTCTGTGGGCTTCA**GCACTTTA**AAGAGGCTGTGTG 3'

C

Oligo Length	Offtargets			
	Exon	Intron	5'UTR	3'UTR
10	308	4887	72	255
11	136	2350	38	119
12	57	1076	17	50
13	17	378	6	12
14	3	198	2	0
15	0	28	1	0
16	0	9	0	0

D

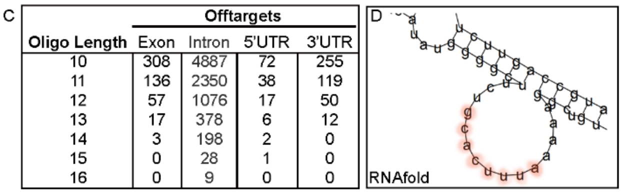

E

Oligo Length	Offtargets			
	Exon	Intron	5'UTR	3'UTR
9	1024	23618	266	937
10	584	14097	155	516
11	230	4997	62	179
12	91	2338	23	78
13	39	1246	13	28
14	21	635	6	6
15	9	252	2	2

F

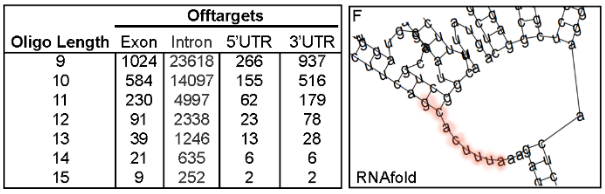

G

Oligo	Length	Offtarget sites (n)	Open (bp)	m△G	h△G
AGCCTTTTTAAAGTGC	16	3	12	-1.06	-8.47
GCCTTTTTAAAGTGCA	16	1	13	-2.42	-11.4
CCTTTTTAAAGTGCAG	16	1	14	-0.11	-4.79
CTTTTTAAAGTGCAGA	16	2	15	-0.06	-4.41
TTTTTAAAGTGCAGAA	16	0	15	-0.03	-4.20
TTTTAAAGTGCAGAAG	16	1	14	-0.05	-4.26
TTTAAAGTGCAGAAGC	16	0	13	-0.22	-4.19
TTAAAGTGCAGAAGCC	16	1	12	-0.59	-4.17
TAAAGTGCAGAAGCCC	16	0	11	-0.73	-4.18

H

Oligo	Length	Offtarget sites (n)	Open (bp)	m△G	h△G
GCCTCTTTAAAGTGC	15	2	8	-0.45	-8.78
CCTCTTTAAAGTGCT	15	6	8	-0.06	-7.00
CTCTTTAAAGTGCTG	15	6	8	-0.04	-6.79
TCTTTAAAGTGCTGA	15	44	8	-0.04	-6.30
CTTTAAAGTGCTGAA	15	41	8	-0.07	-6.23
TTTAAAGTGCTGAAG	15	158	8	-0.10	-5.99
TTAAAGTGCTGAAGC	15	5	7	-0.14	-6.29
TAAAGTGCTGAAGCC	15	3	6	-0.37	-7.61

Supplementary Figure 7. Consideration for design of miR-17 binding motif targeting ASOs. A&B. Genomic sequence of mouse and human *PKD1* 3'UTR is shown with nucleotides highlighted in red indicating the miR-17 binding motif. **A.** The exact region for the mouse-specific *Pkd1* oligo (referred to as P1 oligo in the main text) binding is shown above in purple. **B**. The exact region for the human-specific *PKD1* oligo (referred to as P2 oligo in the main text) binding is shown above in blue. **C&E**. Bioinformatic analysis of proposed oligo lengths and potential off-target binding is noted. The shorter oligonucleotide lengths were predicted to have numerous off-target binding. This analysis for the P1 oligo is shown in C, and P2 is shown in E. **D&F.** RNA fold was used to examine the predicted secondary structure of the *PKD1* 3'-UTR. The nucleotides highlighted in pink comprise the miR-17 binding motif. **G&H.** After determination of optimal length, a tiling approach was used to identify the ASO with the least number of off targets and minimal potential for hair-pinning (monomer delta G, mΔG) or self-dimerization (homodimer delta G, hΔG). The oligos marked with arrows were chosen for further experimentation. The off-target sites are noted in Supplemental tables 2 and 3. The only P1 oligo off target is in the intron of *Ext1* and is not predicted to affect splicing. The P2 oligo off-target sites consist of two uncharacterized noncoding RNAs (LOC107985251 and LOC124901342) and a pseudogene NOX4P1.

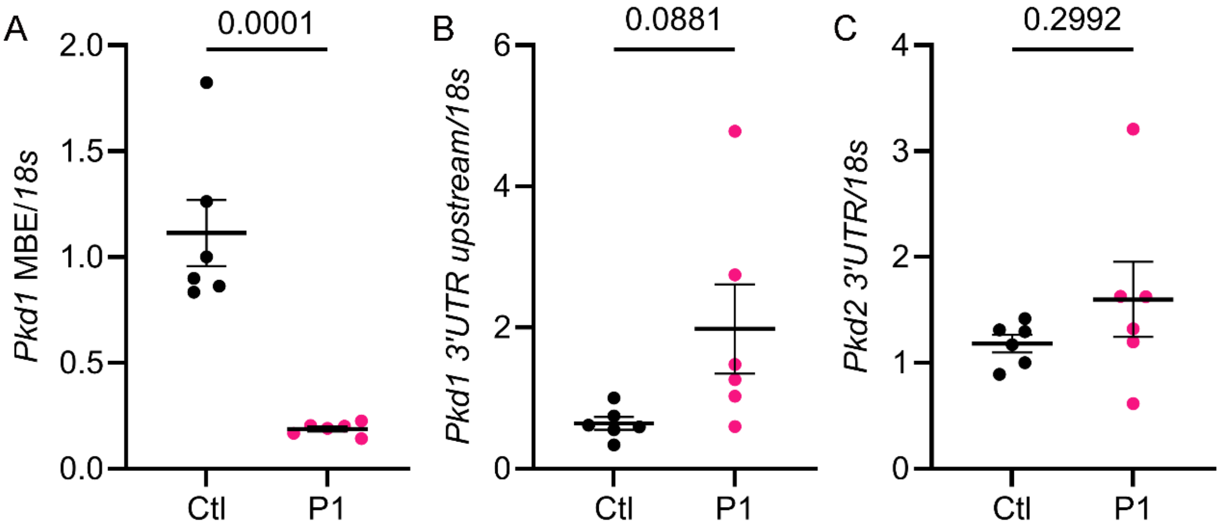

Supplemental Figure 8. P1 oligo specifically binds to the miR-17 motif in *Pkd1* 3'UTR. Murine kidney epithelial cells were transfected with 40 nM P1 oligo or control oligo (Ctl). After 48 hours, cells were harvested and RNA was extracted for target engagement analysis. **A.** qRT-PCR demonstrates no amplification of *Pkd1* mRNA when probed at the *Pkd1*-miR17 motif in cells treated with P1 compared to Ctl oligo, indicating interference due to P1 oligo binding. **B**. However, *Pkd1* mRNA is detected upstream of the oligo binding site, implying specificity of P1 binding to the 3'-UTR motif. **C.** *Pkd2* is direct miR-17 target and contains a conserved miR-17 motif in its 3'UTR. No change in abundance of *Pkd2* mRNA was observed when probed directly at its miR-17 binding site, indicating specific binding of P1 to *Pkd1* 3-UTR but not to the closely related *Pkd2* 3'UTR. Error bars indicate SEM. N=6 biological replicates in all group. Statistics: Unpaired, two-tailed, t-test.

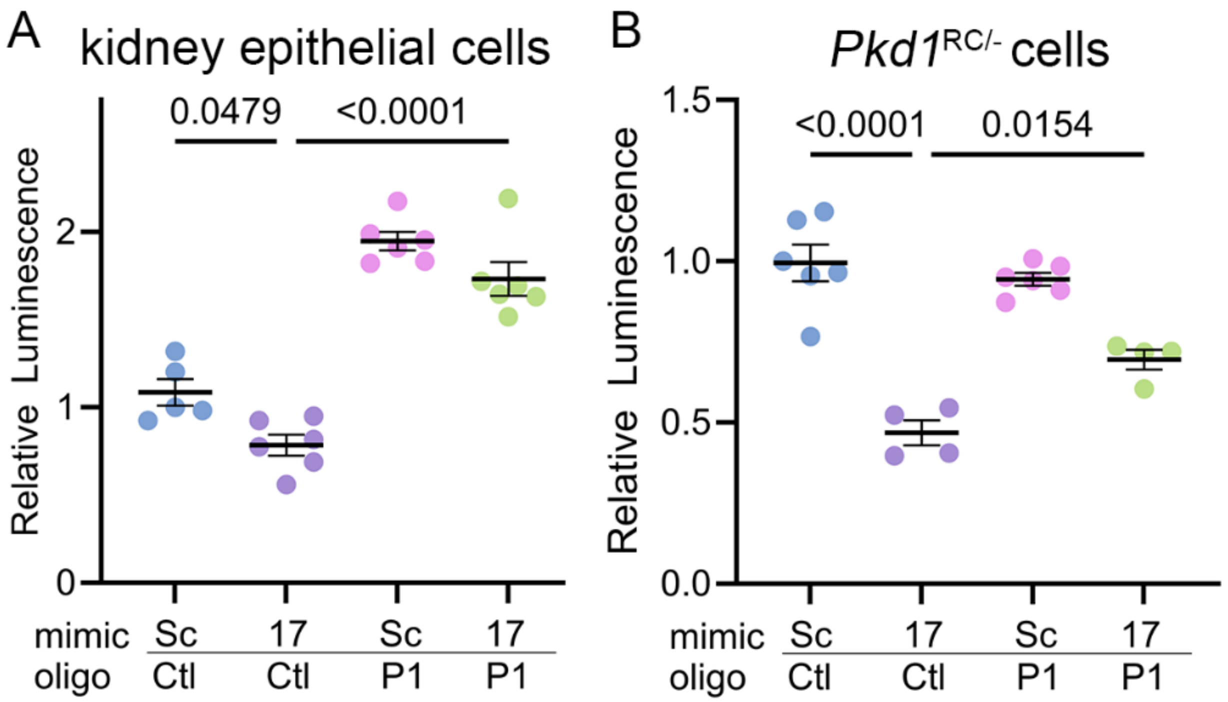

Supplemental Figure 9. Functional engagement of the *Pkd1* 3'UTR by P1 oligo. A&B. Murine kidney epithelial cell lines were transfected with *Pkd1* 3'UTR luciferase reporter plasmid, miR-17 or scramble mimic, and P1 or control oligo. Luciferase reporter assay demonstrates repression of luminescence in the setting of miR-17 mimic which is recovered in the presence of P1 oligo. Error bars indicate SEM. Each data point is biological replicate with an average of 3 technical replicates. Statistics: ANOVA with Tukey's.

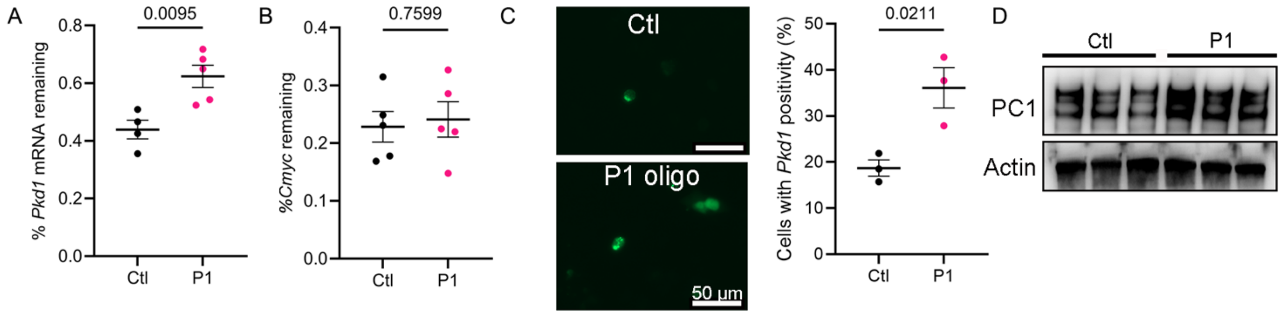

Supplemental Figure 10. P1 oligo stabilizes endogenous *Pkd1* mRNA and increases PC1 protein. **A-B**. Collecting duct-derived kidney epithelial cells treated with control (ctl) oligo or P1 oligo for 48 hours and were subsequently treated with Actinomycin D for 6 hours to arrest de-novo transcription. **A**. qRT-PCR demonstrates 30% more *Pkd1* mRNA in cells treated with P1 oligo compared to control oligo treated cells, implying improved *Pkd1* transcript stability in P1-treated cells. **B.** As a negative control, the abundance of c-Myc mRNA remains unchanged**.** **C.** Collecting duct-derived kidney epithelial cells treated with control (ctl) oligo or P1 oligo for 48 hours and co-transfected with csm-GFP-*Pkd*1-SgRNA system for live-cell detection of *Pkd1* mRNA transcript. Immunofluorescence images and subsequent quantification show that cells treated P1 oligo exhibit increased *Pkd1* mRNA signal compared to Ctl-oligo treated cells. **D.** Western blot shows PC1 protein is increased in P1-treated kidney epithelial cells compared to Ctl-oligo treated cells. Error bars indicate SEM. Statistics: Unpaired, two-tailed, t-test. Actin serves as the loading control.

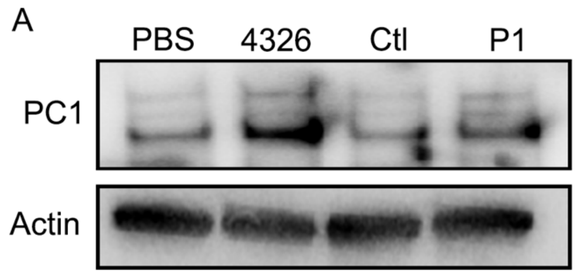

Supplemental Figure 11. Anti-miR-17 compound RGLS4326 and P1 oligo both raise PC1. A. *Pkd1*$^{RC/-}$ cells were treated with either RGLS4326 or P1 oligo and their respective controls (PBS or Ctl oligo) for 48 hours. Immunoblot reveals that RGLS4326 and P1 oligo both raise PC1 protein.
